# The French national prospective cohort of patients co-infected with HIV and HCV (ANRS CO13 HEPAVIH): Early findings, 2006-2010

**DOI:** 10.1186/1471-2334-10-303

**Published:** 2010-10-22

**Authors:** Marc-Arthur Loko, Dominique Salmon, Patrizia Carrieri, Maria Winnock, Marion Mora, Laurence Merchadou, Stéphanie Gillet, Elodie Pambrun, Jean Delaune, Marc-Antoine Valantin, Isabelle Poizot-Martin, Didier Neau, Philippe Bonnard, Eric Rosenthal, Karl Barange, Philippe Morlat, Karine Lacombe, Anne Gervais, François Rouges, Alain Bicart See, Caroline Lascoux-Combe, Daniel Vittecoq, Cécile Goujard, Claudine Duvivier, Bruno Spire, Jacques Izopet, Philippe Sogni, Lawrence Serfaty, Yves Benhamou, Firouzé Bani-Sadr, François Dabis

**Affiliations:** 1 INSERM, U897 and ISPED, Université Victor Segalen, Bordeaux, France; 2 Service des Maladies Infectieuses et Tropicales, Hôpital Cochin, AP-HP, Paris, France; 3 Université Paris Descartes, Paris, France; 4 INSERM, U912 (SE4S), Marseille, France; 5 Université Aix Marseille, IRD, UMR-S912, Marseille, France; 6 ORS PACA, Observatoire Régional de la Santé Provence Alpes Côte d'Azur, Marseille, France; 7 Service des Maladies Infectieuses et Tropicales, Hôpital Pitié Salpétrière, AP-HP, Paris, France; 8 INSERM, U943, Paris, France; 9 Service d'Immuno-Hématologie Clinique/CISIH, Hôpital Sainte Marguerite, Marseille, France; 10 Service des Maladies Infectieuses et Tropicales, Hôpital Pellegrin, Bordeaux, France; 11 Service des Maladies Infectieuses et Tropicales, Hôpital Tenon, AP-HP, Paris, France; 12 Service de Médecine Interne, Hôpital de l'Archet, Nice, France; 13 Service d'Hépato-Gastro-Entérologie, Hôpital Purpan, Toulouse, France; 14 Service de Médecine Interne et Maladies Infectieuses, Hôpital Saint-André, Bordeaux, France; 15 Service des Maladies Infectieuses et Tropicales, Hôpital Saint-Antoine, Paris, France; 16 Service des Maladies Infectieuses et Tropicales, Hôpital Bichat Claude Bernard, Paris, France; 17 Service de Médecine Interne - Unité VIH, Hôpital Avicenne, AP-HP, Bobigny, France; 18 Service de Médecine, Hôpital Joseph Ducuing, Toulouse, France; 19 Service de Médecine Interne, Hôpital Saint-Louis, AP-HP, Paris, France; 20 Service des Maladies Infectieuses et Tropicales, Hôpital Paul Brousse, AP-HP, Paris, France; 21 Service des Maladies Infectieuses et Tropicales, Hôpital Bicêtre, AP-HP, Paris, France; 22 Service des Maladies Infectieuses et Tropicales, Groupe Hospitalier Necker-Enfants Malades, AP-HP, Paris, France; 23 Institut Fédératif de Biologie, Laboratoire de Virologie, Hôpital Purpan, Toulouse, France; 24 Unité d'Hépatologie, Hôpital Cochin, AP-HP, Paris, France; 25 Service d'Hépato-Gastro-Entérologie, Hôpital St-Antoine, AP-HP, Paris, France; 26 Service d'Hépato-Gastro-Entérologie, Hôpital Pitié Salpétrière, AP-HP, Paris, France

## Abstract

**Background:**

In France, it is estimated that 24% of HIV-infected patients are also infected with HCV. Longitudinal studies addressing clinical and public health questions related to HIV-HCV co-infection (HIV-HCV clinical progression and its determinants including genetic dimension, patients' experience with these two diseases and their treatments) are limited. The ANRS CO 13 HEPAVIH cohort was set up to explore these critical questions.

To describe the cohort aims and organization, monitoring and data collection procedures, baseline characteristics, as well as follow-up findings to date.

**Methods:**

Inclusion criteria in the cohort were: age > 18 years, HIV-1 infection, chronic hepatitis C virus (HCV) infection or sustained response to HCV treatment. A standardized medical questionnaire collecting socio-demographic, clinical, biological, therapeutic, histological, ultrasound and endoscopic data is administered at enrolment, then every six months for cirrhotic patients or yearly for non-cirrhotic patients. Also, a self-administered questionnaire documenting socio-behavioral data and adherence to HIV and/or HCV treatments is administered at enrolment and yearly thereafter.

**Results:**

A total of 1,175 patients were included from January 2006 to December 2008. Their median age at enrolment was 45 years and 70.2% were male. The median CD4 cell count was 442 (IQR: 304-633) cells/μl and HIV RNA plasma viral load was undetectable in 68.8%. Most participants (71.6%) were on HAART. Among the 1,048 HIV-HCV chronically co-infected patients, HCV genotype 1 was predominant (56%) and cirrhosis was present in 25%. As of January, 2010, after a median follow-up of 16.7 months (IQR: 11.3-25.3), 13 new cases of decompensated cirrhosis, nine hepatocellular carcinomas and 20 HCV-related deaths were reported, resulting in a cumulative HCV-related severe event rate of 1.9/100 person-years (95% CI: 1.3-2.5). The rate of HCV-related severe events was higher in cirrhotic patients and those with a low CD4 cells count, but did not differ according to sex, age, alcohol consumption, CDC clinical stage or HCV status.

**Conclusion:**

The ANRS CO 13 HEPAVIH is a nation-wide cohort using a large network of HIV treatment, infectious diseases and internal medicine clinics in France, and thus is highly representative of the French population living with these two viruses and in care.

## Background

Hepatitis C virus (HCV) infection is common among the population living with HIV. In France, it is estimated that 24% of HIV-infected patients are also infected with HCV [1]. There is evidence that HIV negatively affects the natural history of HCV infection, with an accelerated progression of HCV-related liver disease towards cirrhosis, decompensated liver disease or hepatocellular carcinoma [2-4]. In contrast, there are conflicting data about the impact of HCV infection on the course of HIV disease [5-7].

Since the advent of highly active antiretroviral therapy (HAART), which inhibits HIV replication and thus enables patients to reach and sustain an effective immune response, the mortality due to opportunistic infections in HIV-infected patients has been drastically reduced. As a consequence, chronic liver disease has emerged as a major cause of morbidity and mortality among HIV-infected individuals [8,9]. In addition, the risk of hepatotoxicity linked to antiretroviral drugs and alcohol abuse is increased in subjects with underlying HCV infection [10].

While the use of pegylated interferon and ribavirin has increased the chances of clearing HCV infection mainly in patients presenting HCV genotypes 2 or 3 [11,12], this treatment has also been shown to significantly increase the burden of perceived side effects in patients receiving HAART [13]. This is of utmost importance as anti-HCV treatment and HAART require sustained high levels of adherence in order to be effective.

To date, longitudinal studies on factors associated to clinical progression of HIV-HCV co-infection, as well as those related to patients' experience with these two diseases and their treatments, are limited [14-17].

Within this framework, the French National Agency for Research on AIDS and Viral hepatitis (ANRS) CO 13 HEPAVIH collaboration initiated in 2005 a closed, prospective, hospital-based cohort to address clinical and public health issues around HIV-HCV co-infection, which are summarized in the main objectives of the cohort, as follows:

1) In the short-term (1-2 years after end of enrolment): a) to describe HIV-HCV co-infection in France and its management, including socio-behavioral, diagnostic and therapeutic aspects, b) to validate the accuracy of non-invasive methods for assessing liver fibrosis;

2) In the mid-term (3-4 years): a) to study the impact of HCV treatment on the clinical and immuno-virological response of HIV, b) to study the pharmacokinetics of antiretroviral drugs and anti-HCV drugs according to histological liver disease stage, and interactions between antiretroviral and anti-HCV drugs, c) to identify physicians' and patients' barriers to access to HCV treatment and adherence to HIV or HCV treatments;

3) In a longer-term (5 years and beyond): a) to describe the course of HIV-HCV co-infection and to assess the role of factors such as clinical characteristics and behaviors, genetics, exposure to HCV treatment, HIV immuno-virolological status, HIV clinical events and HAART use, on the occurrence of a decompensated liver disease or hepatocellular carcinoma, b) to study the impact of antiretroviral drugs on the progression of liver fibrosis, c) to identify predictors of patients' health-related quality of life and perceived side effects and their evolution.

The aim of this article is to describe this ongoing cohort, the characteristics of patients at enrolment as well as the main follow-up achievements to date.

## Methods

### Inclusion criteria

Consecutive patients seen in 17 hospital wards between January, 2006 and December, 2008 and who fulfilled the following criteria were enrolled in the ANRS CO 13 HEPAVIH cohort: aged 18 years or more, HIV-1/HCV chronically co-infected as confirmed by a positive HIV antibody test and an HCV RNA assay (regardless of the clinical stage, gender or transmission group), written informed consent.

In addition to these chronically co-infected patients, HIV/HCV co-infected patients who had cleared their HCV after a successful anti-HCV therapy, as evaluated by a negative HCV RNA six months after the end of anti-HCV treatment could be included. Thus, at their enrolment, these patients were characterized as HCV sustained responders.

Patients on HCV treatment at enrolment, with undetectable HCV-RNA but not yet classified as sustained responders were included in the cohort at the end of their treatment phase.

### Schedule of follow-up

The schedule of follow-up visits is based on clinical practice as recommended by the European consensus conferences on hepatitis C, that is every six months for cirrhotic patients and every year for non-cirrhotic patients [18-20]. Five specific additional visits are scheduled when anti-HCV treatment is initiated (baseline visit, then at month 1, 3, at the end of treatment and six months after the end of treatment).

A patient self-administered questionnaire is scheduled at enrolment and yearly thereafter.

### Data collection

A standardized medical questionnaire is completed by physicians at each clinical visit. Patients also use self-administered questionnaires.

#### Clinical and histological outcomes

**Figure 1:**
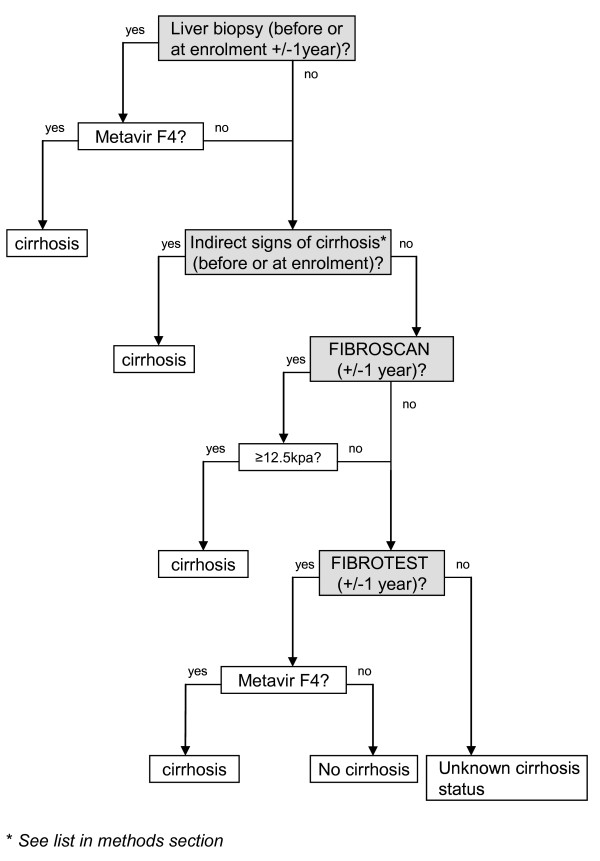
**Algorithm for assessing cirrhosis, ANRS CO 13 HEPAVIH cohort of HIV-HCV co-infected patients**.

Patients have a regular physical examination. The results of liver biopsy (LB), whenever available, are documented and graded with the Metavir system. In all cases, a systematic assessment of liver fibrosis is also recommended using non-invasive methods (FibroTest^™ ^and/or FibroScan^™^) [21,22]. FibroScan cut-offs used for the conversion in the Metavir score are: F0-F1: <7.1 Kpa, F2: 7.1-9.5 Kpa, F3: 9.5-12.5 Kpa, F4: ≥12.5 Kpa [23]. Given the discordances between liver biopsy results and those of non-invasive methods, cirrhosis is diagnosed based on a hierarchical algorithm (Figure 1). This algorithm considers first the result of LB (F4 in the METAVIR system), then the presence of indirect clinical signs of cirrhosis (ascites, esophageal varices with or without bleeding, hepatic encephalopathy), followed by the FibroScan^™ ^result (with an elasticity value >12.5 KPa), and lastly the result of FibroTest^™ ^(F4 in the METAVIR system). A final confirmation of the diagnosis obtained with this algorithm is made by the physician in charge of the patient. The quality criteria of LB (at least six portal tracts), FibroScan^™ ^(success rate ≥60% or an interquartile range [IQR] less than 30% of the median liver stiffness measurement [LSM] value) and FibroTest^™ ^(absence of atazanavir or indinavir-related hyperbilirubinemia) are taken into consideration when using the algorithm.

An ultrasound examination is performed to assess possible complications of the liver disease. In case of suspected or diagnosed cirrhosis, endoscopic examination is also prescribed.

All these data are collected at enrolment and yearly (every six months for cirrhotic patients) and are combined to define different indicators of HCV disease progression.

Moreover, data on mortality, HIV- and HCV-related clinical events with special focus on the end-stage liver disease and hepatocellular carcinoma, access to HCV treatment, and responses to anti-HCV and HIV treatments are also studied as outcomes.

#### Socio-behavioral outcomes

Together with clinical outcomes, adherence to HIV or HCV treatments or patients' quality of life as expressed by the WHO-QOL (World Health Organization Quality Of Life for HIV) [24] and the fatigue impact scales are investigated.

#### Exposure factors or potential predictors of outcomes

Clinical and biological variables are studied as potential predictors of HCV progression.

Presence of lipodystrophy is considered when a lipoatrophy or a lipohypertrophy is identified, whatever the anatomical site.

Blood tests performed at enrolment and during follow-up cover aspects of general health: haemoglobin and platelet count, biological markers of hepatitis C virus infection, markers indicating past or current infection with hepatitis B virus and parameters indicative of the progression of HIV infection (CD4 cells count, HIV plasma RNA). Other biological tests such as liver function tests, total cholesterol, HDL and LDL-cholesterol, triglycerides, creatinin, glycemia, insulinemia, lactates, lipase, apolipoprotein A1, haptoglobin, α2-macroglobulin, cryoglobulin and complement components (CH50, C4) are also performed at enrolment and during follow-up. Insulin resistance (IR) is measured using the homeostasis model assessment of IR (HOMA) [25].

Patients' socio-demographic and psychosocial characteristics, alcohol and substance use, perceptions about treatments and their efficacy, use of psychotropic medications or substitution treatments, self-reported symptoms, depressive symptoms (CES-D scale [Center for Epidemiologic Studies Depression Scale]) [26] and anger (STAXI II scale [State-Trait Anger Expression Inventory]) are used as potential explanatory variables for clinical and socio-behavioral outcomes. Physicians' socio-demographic characteristics and data on their professional experience, their opinion about their patients' current drug and alcohol use as well as their perception of the patients' adherence to scheduled visits and treatment(s) are also used as potential explanatory variables for clinical and socio-behavioral outcomes.

Blood samples (plasma, serum and whole blood) are collected every year, as well as during anti-HCV treatment, to be stored centrally.

### Data management

Clinical and biological questionnaires are collected and centralized at the INSERM U897 Epidemiology centre in Bordeaux, France, where they are checked for accuracy and completeness. Self-administered questionnaires are sent to the INSERM U912 Social Sciences Unit in Marseille where they are validated and then double-entered in a specific database. Standardized procedures are used to compute the scores of the administered scales (WHO-QOL, CES-D, STAXI-II, FIS). A unique database merging clinical, socio-behavioral and psychosocial data is built every six months for all statistical analyses.

The database used for the current manuscript included information recorded at enrolment as well as during follow-up up to January 2010.

### Statistical Analyses

Continuous variables are described by their median value and interquartile range (IQR); categorical variables are described as percentages.

Event rates are compared using a Poisson regression analysis. Survival curves are compared using the Log-rank test.

All statistical tests are two-sided, with a type I error of 5%.

### Ethical aspects

The study was designed and performed in accordance with the Declaration of Helsinki and was approved by the ethics committee of Cochin university hospital in Paris.

## Results

### Patients' characteristics

From January 2006, until December 2008 (enrolment period), a total of 1,175 patients were enrolled in the cohort, 1,048 HIV-HCV chronically co-infected patients and 127 HCV sustained responders.

Table 1 summarizes the baseline characteristics of the 1,175 enrolled patients and Tables 2 and 3 provide further HIV and HCV-related characteristics. Their median age was 45 years (IQR: 42-48 years) and most participants were males (70.2%).The median baseline CD4 absolute count was 442 cells/μl (IQR: 304-633) and HIV RNA plasma viral load was undetectable in 68.8% of the patients.

**Table 1:** Baseline socio-demographic, clinical and biological characteristics of patients included in the ANRS CO 13 HEPAVIH cohort of HIV-HCV co-infected patients, France, 2006-2008

	Total N=1175	HCV chronically infected patients N=1048	HCV sustained responders N=127
Age (years), median (IQR)	45 (42-48)	45 (42-48)	46 (43-49)
Male gender, N (%)	825 (70.2)	735 (70.1)	90 (70.9)
BMI (kg/m^2^), median (IQR)	21.6 (19.7-24.1)	21.6 (19.7-24.1)	22.0 (20.1-24.3)
High school certificate, N (%)	302(34)	260 (33)	42 (42)
Employment, N (%)	481 (49)	423 (48)	58 (52)
Quality of life (WHOQOL), median (IQR)			
Physical	14 (11-16)	14 (11-16)	14 (11-16)
Psychological	14 (12-17)	14 (12-17)	14 (12-16)
Level of independence	14 (11-17)	14 (11-17)	14 (10-17)
Social relationship	14 (12-17)	14 (11-17)	15 (12-17)
Environment	15 (13-17)	15 (12-17)	15 (14-17)
Spirituality/religion	14 (12-16)	14 (12-16)	14 (12-17)
Alcohol consumption (gr/day): >20 (women) or >30 (men), N (%)	120 (10.7)	109 (10.9)	11 (8.9)
Daily cocaine use in the last month, N (%)	136 (14)	123 (15)	13 (13)
Depressive symptoms (CES-D>17 for men, CES-D>23 for women), N (%)	379 (41)	343 (41)	36 (34)
HOMA, median (IQR)	2.0 (1.3-3.4)	2.1 (1.3-3.5)	2.1 (1.4-3.1)
Lipodystrophy, N (%)	635 (54.0)	560 (53.4)	75 (59.1)
Diabetes, N (%)	69 (5.9)	62 (5.9)	7 (5.5)
ALT (x normal), median (IQR)	1.2 (0.8-2.0)	1.3 (0.9-2.1)	0.6 (0.4-0.8)
AST (x normal), median (IQR)	1.1 (0.8-1.8)	1.2 (0.8-1.9)	0.7 (0.5-0.9)
Hepatitis B surface antigen positive, N (%)	22 (2.0)	20 (2.0)	2 (1.7)

BMI: body mass index; HOMA : homeostasis model assessment of insulin-resistance; IQR: interquartile range; CES-D : Center for Epidemiologic Studies Depression scale; WHO-QOL : World Health Organization Quality Of Life for HIV

**Table 2:** Baseline characteristics of HIV infection of patients included in the ANRS CO 13 HEPAVIH cohort of HIV-HCV co-infected patients, France, 2006-2008

	Total N=1175	HCV chronically infected patients N=1048	Sustained HCV responders N=127
CDC clinical stage: C, N (%)	324 (27.8)	300 (28.9)	24 (19.2)
Years since HIV diagnosis, median (IQR)	18 (13-20)	18 (13-20)	18 (14-21)
HIV transmission category, N (%)			
Intravenous drug use	724 (62.0)	651 (62.5)	73 (57.9)
Sexual	318 (27.2)	282 (27.1)	36 (28.6)
Transfusion	85 (7.3)	73 (7.0)	12 (9.5)
Other or unknown	40 (3.4)	35 (3.4)	5 (4.0)
Nadir CD4 (/μl), median (IQR)	150 (66-248)	146 (65-247)	169 (96-251)
CD4 cells count at initiation of antiretroviral treatment (/μl), median (IQR)	242 (139-357)	240 (132-354)	256 (191-396)
CD4 cells count at baseline (/μl), median (IQR)	442 (304-633)	438 (300-630)	492 (345-662)
CD4 cells count at baseline (/μl), N (%)			
<200	130 (11.2)	126 (12.2)	4 (3.1)
200-350	251 (21.6)	223 (21.6)	28 (22.0)
351-500	301 (25.9)	269 (26.1)	32 (25.2)
>500	479 (41.3)	416 (40.2)	63 (49.6)
Patients with <40 HIV RNA copies/ml, N (%)	797 (68.8)	702 (68.1)	95 (74.8)
Patients receiving HAART, N (%)	841 (71.6)	750 (71.6)	91 (71.6)
2 NRTI + 1 PI	613 (52.2)	552 (52.7)	61 (48.0)
2 NRTI + 1 NNRTI	183 (15.6)	158 (15.1)	25 (19.7)
1 NRTI + 1 NNRTI + 1 PI	45 (3.8)	40 (3.8)	5 (3.9)
Regimen including raltegravir	26 (2.2)	23 (2.2)	3 (2.4)

PI: protease inhibitor; NRTI: nucleoside reverse transcriptase inhibitor; NNRTI: non-nucleoside reverse transcriptase inhibitor; IQR: interquartile range;HAART: highly active antiretroviral therapy

**Table 3:** Baseline characteristics of HCV infection of patients included in the ANRS CO 13 HEPAVIH cohort of HIV-HCV co-infected patients, France, 2006-2008

	Total N=1175	HCV chronically infected patients N=1048	Sustained HCV responders N=127
Years since HCV diagnosis, median (IQR)	-	10 (6-14)	-
HCV genotype, N (%)	-		-
1		577 (56.0)	
2		39 (3.8)	
3		187 (18.1)	
4		225 (21.8)	
5 or other		3 (0.3)	
HCV plasma RNA (Log_10_) (IU/ML), median (IQR)	-	6.2 (5.7-6.5)	-
Past anti HCV therapy, N (%)			
Interferon alone	40 (3.4)	33 (3.1)	7 (5.5)
Interferon+ ribavirin	93 (7.9)	65 (6.2)	28 (22)
Pegylated interferon plus ribavirin	365 (31.1)	273 (26.0)	92 (72.5)
Cirrhosis*, N (%)	255 (25.2)	229 (25.0)	26 (26.8)
Documented decompensated liver disease, N (%)	32 (2.7)	27 (2.6)	5 (3.9)

* *according to the study algorithm of cirrhosis (see Figure*1*)*

For HIV-HCV chronically co-infected patients, the median time elapsed since the diagnosis of HCV infection was 10 years (IQR: 6-14) and the main source of contamination was intravenous drug exposure. HCV genotype 1 was predominant (56%). The median HCV RNA plasma viral load was 6.2 logUI/ml (IQR: 5.7-6.5).

**Figure 2:**
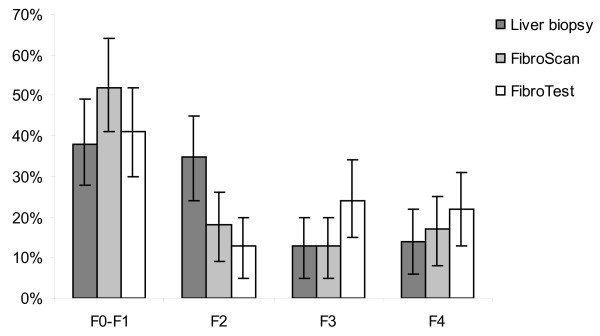
**Prevalence of liver fibrosis and its 95% confidence interval at baseline (+/- 1 year) in patients with concomitant results of liver biopsy, FibroScan^™ ^and FibroTest^™^, and regardless of HCV status (N = 78), ANRS CO 13 HEPAVIH cohort of HIV-HCV co-infected patients, France, 2006-2008**.

Evaluation of liver fibrosis at enrolment (+/- one year), with LB, FibroTest^™ ^or FibroScan^™ ^was available for 196, 732 and 824 patients, respectively. In patients for whom the three examinations were available (N = 78), the distribution of fibrosis stages was different depending on the test used. The proportion of patients with F3 or F4 stage was higher with FibroTest^™ ^(46%) than with FibroScan^™ ^(30%) or LB (27%), whereas the proportion of patients with F0-F1 stage was higher with FibroScan^™ ^(52%), compared to FibroTest^™ ^(41%) and LB (38%) (Figure 2). Twenty-five percent of patients were documented as cirrhotic at their enrolment, using the study algorithm of cirrhosis and confirmation of diagnosis by physicians.

At enrolment, 71.6% of the patients were receiving HAART, for a median duration of 9.3 years (IQR: 4.6-12.1). The most frequent drug combination was two nucleoside reverse transcriptase inhibitors (NRTI) and one ritonavir-boosted protease inhibitor (PI), representing 57.6% of the treated patients whereas the combination of two NRTIs and one non nucleoside reverse transcriptase inhibitor (NNRTI) represented 17.2% of treated patients (Table 2).

With regards to anti-HCV therapy, 371 (35.4%) HIV-HCV chronically infected patients had been treated at least once for HCV before enrolment in the cohort, with the following regimens: interferon alone: n = 33, interferon + ribavirin: n = 65, pegylated interferon + ribavirin: n = 273 (Table 3).

Since enrolment up to January, 2010, 238 patients received pegylated interferon plus ribavirin therapy: 74 among those previously treated patients and 164 among treatment-naïve patients. Hence, overall a total of 535 HIV-HCV chronically infected patients (51%) had access at least once to anti-HCV therapy up to the closing date of the present report.

Among the 238 patients treated for HCV infection since enrolment in the cohort, 127 (53%) so far, had available data on sustained virological response (SVR). Overall, 40 (31.5%) achieved sustained virological response. The rate of HCV clearance was 34% in treatment-naïve patients and 27% in the previously treated group. When stratified according to HCV genotypes, the rate of SVR was 20% in patients with genotype 1, 62% in patients with genotype 2 or 3 and 36% in patients with genotype 4.

### Main clinical events occurring since enrolment and time-to-event analyses

Overall, the cumulative follow-up duration, calculated between the date of enrolment of each patient and the last follow-up visit, the date of consent removal or the date of death was 1,775 person-years (P-A). The median duration of follow-up was 16.7 months (IQR: 11.3-25.3).

Up to January 2010, 13 new cases of decompensated cirrhosis (presence of ascites, varices bleeding, or hepatic encephalopathy) were diagnosed, all in the HIV-HCV chronically infected patients. Nine new cases of hepatocellular carcinoma (six in HIV-HCV chronically infected patients and three in sustained responders) were reported in the cohort. Forty-nine deaths (47 in HIV-HCV chronically infected patients and two in sustained responders) occurred and 20 (41%) of them were categorized as attributable to HCV infection.

**Figure 3:**
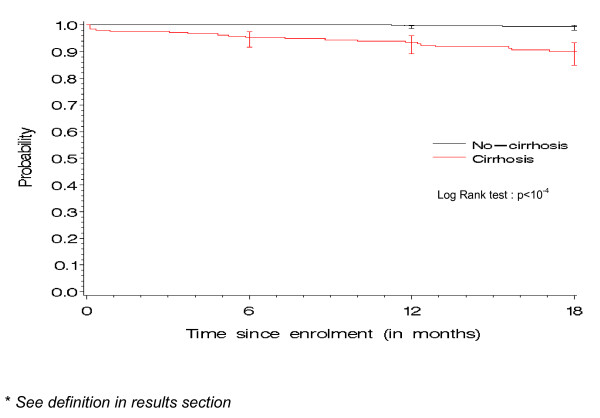
Probability of remaining free of HCV-related severe event*, according to cirrhosis status, ANRS CO 13 HEPAVIH cohort of HIV-HCV co-infected patients. Bars represent confidence intervals at 6, 12 and 18 months

A time-to-event analysis was performed to estimate the incidence since enrolment, of HCV-related severe events, defined as the occurrence of a first hepatic decompensation (presence of ascites, varices bleeding, or hepatic encephalopathy), hepatocellular carcinoma or HCV-related death. As of January, 2010, a total of 33 HCV-related severe events (30 in HIV-HCV chronically infected patients and three in sustained responders) were reported, resulting in a cumulative HCV-related severe event rate of 1.9/100P-A (95% CI: 1.3-2.5). When stratified according to cirrhosis status, the rate of HCV severe events was higher for cirrhotic patients (6.9/100P-A) than for non-cirrhotic patients (0.35/100P-A) (p < 10^-4^) (Figure 3). The rate of HCV-related severe events was also higher in patient with a baseline CD4 absolute count <200/μl than in those with a CD4 count above this threshold (5.5 vs 1.4/100P-A; p = 0.002). The rate of HCV-related severe events did not differ according to other factors such as sex (2.1 in male vs 1.4/100P-A in female; p = 0.32), age (1.7 in patients aged 45 years or less vs 2.1/100P-A in others; p = 0.55), alcohol consumption (2.9 if alcohol consumption >20 gr/day [women] or >30 gr/day [men] vs 1.8/100P-A in others; p = 0.36), CDC clinical stage (2.8 in patients with CDC clinical stage C vs 1.6/100P-A in others; p = 0.14) or HCV status (1.9 in HIV-HCV chronically infected patients vs 1.8/100P-A in HCV sustained responders; p = 0.88).

Given the algorithm used to identify cirrhotic patients, the performance of this algorithm, as well as of each contributing test (LB, FibroScan, FibroTest) on the occurrence of HCV-related severe events was evaluated. The negative predictive value (NPV) of the algorithm and of LB, FibroScan and FibroTest for predicting the absence of HCV-related severe events was good (98-99%). However, the positive predictive values were low: 12.5% for the algorithm and 7.4 to 10.3% for the individual tests (Table 4).

**Table 4:** Predictive values of the cirrhosis algorithm and of liver biopsy, FibroScan and FibroTest for the prediction of HCV-related severe events occurrence, ANRS CO 13 HEPAVIH cohort of HIV-HCV co-infected patients

		HCV-related severe events^a^			
				
		Absence (n)	Presence (n)	PPV (%)	NPV (%)
Algorithm* N=987	Absence of cirrhosis	752	4	12.5	99.5
	Presence of cirrhosis	203	29		
Liver Biopsy N=192	Metavir < F4	161	2	10.3	98.8
	Metavir F4	26	3		
FibroScan N=806	<12.5KPa	653	4	7.4	99.4
	≤ 12.5 KPa	138	11		
FibroTest N=718	Metavir < F4	528	7	8.2	98.7
	Metavir F4	168	15		

* See definition in Methods section; PPV: positive predictive value; NPV negative predictive value

*a HCV-related severe events*: ascites, varices bleeding, hepatic encephalopathy, *hepatocellular carcinoma or HCV-related death*

### Socio-behavioral findings

Using data from the medical and self-administrated questionnaires, we showed that successful treatment for depression may reduce the negative impact that fatigue has on cognitive, physical and social functioning of HIV-HCV co-infected patients. Indeed, this analysis published elsewhere has demonstrated that the impact of fatigue on daily functioning in HIV-HCV co-infected patients is determined by two dimensions; on one hand, psychosocial and demographic factors as expressed by the number of discomfort side effects, employment status and gender (women), on the other hand, clinical factors related to the severity of HIV disease, choice of antiretroviral drugs, depression and its management [27].

## Discussion

The ANRS CO 13 HEPAVIH cohort has been assembled through a unique nation-wide collaboration of HIV treatment, infectious diseases, internal medicine and hepatology centres in France. This article provides information on the objectives of this cohort, the monitoring and data collection procedures, the baseline characteristics of patients, as well as the main follow-up findings to date.

A total of 1,175 patients were enrolled in the cohort, including 1,048 HIV-HCV chronically co-infected patients. Compared to results of a national cross-sectional survey on French HIV-HCV co-infected patients performed in 2001 [28], the ANRS CO 13 HEPAVIH HIV-HCV co-infected patients tended to be older at enrolment (mean age 45 vs. 39 years). They were also less often in CDC clinical stage C (28% vs. 34%) [29] and had a higher CD4 cells count (CD4 > 500 cells/μl :41% vs. 30%) than patients surveyed in 2001. These differences could be due to an improved control of HIV disease over time through the use of more effective antiretroviral treatment regimens. These differences were also observed when our cohort was compared to cohorts from other countries, such as the Swiss HIV cohort (median age 35 years, median CD4 172/mm^3^) [6]. However, our patients were comparable to those included in a cross-sectional national study performed in 2006 in France [30]. Also, the frequency of patients in our cohort with histological cirrhosis was similar to that reported in other studies [1,28].

HCV genotypes 1 and 4 were mostly observed in HIV-HCV chronically co-infected patients (56% and 22%, respectively), which was consistent with data from other hospital-based cohorts in France and elsewhere in Europe [14,31,32]. This high prevalence of genotypes 1 and 4 in the HIV-HCV chronically co-infected patients of our cohort could be due to the recruitment of relapsers or non-responders to a previous course of HCV therapy. Indeed, in those relapsers or non-responders, HCV genotypes 1 and 4 are highly prevalent, contrary to HCV genotypes 2 and 3 [11,33].

Fifty-one percent of HIV-HCV chronically infected patients in the ANRS CO 13 HEPAVIH cohort had access at least once to anti-HCV therapy. This proportion was higher than in previous reports in France (with only 31 to 45% of patients treated for HCV infection) [1,28], suggesting an improvement in the management of HIV-HCV patients over time. This relatively good uptake of HCV therapy in our cohort also contrasted with rates reported in HIV-HCV patients in Spain so far (41%) and with low rates observed in studies conducted in North America (6-33%) [32,34-36]. Differences in patients' characteristics and easy access to medication (including free or cheap provision of drugs) may explain at least partly these differences.

During follow-up, 33 HCV-related severe events occurred after a median follow-up de 16.7 months. As expected, the incidence of these events was higher in cirrhotic patients, emphasizing the importance to accurately diagnose cirrhosis in such patients. In our cohort, an algorithm combining liver biopsy, Fibrotest and Fibroscan was used to identify cirrhotic patients, who are thus likely to benefit from a regular follow-up. The negative predictive value of this algorithm for predicting the absence of HCV-related severe events was good (99.5%). However its positive predictive value was low (12.5%), probably due to the relatively short follow-up duration in the cohort. Nevertheless, this algorithm had higher predictive values than the individual tests for the prediction of HCV-related severe events. This finding suggests that the use of an algorithm could lead to an acceptable rate of misclassification for cirrhosis, thus allowing a better case management. The main limitation of this algorithm is the quality criterion of liver biopsy (at least six portal tracts) which may be insufficient to accurately diagnose cirrhosis. However, only 196 (16.7%) patients had a liver biopsy during the year preceding or following inclusion in the cohort. In addition, given the hierarchical dimension of this algorithm, it is likely that patients inaccurately diagnosed by liver biopsy may then be correctly diagnosed by the other tests, thus minimizing the difficulties of performing and interpreting liver biopsy in such patients.

The participants enrolled in the ANRS CO 13 HEPAVIH cohort came from 17 clinical centres, representing four regions of high prevalence for HCV infection in France. Despite this relatively limited geographical distribution, our sample seemed comparable to other national multicentric studies in France. Therefore, the ANRS CO 13 HEPAVIH cohort mirrors the whole French population living with HIV and HCV and currently in care, thus providing a good representativeness of co-infection in real life situations.

The main strength of the ANRS CO 13 HEPAVIH cohort is the combined longitudinal data collection of clinical and socio-behavioral items that makes this cohort unique in the field. This organization provides not only the possibility to study risk factors of HCV progression but also to monitor both patient's response and experience with treatment and to investigate the multi-factorial reasons for non-treatment and also non-response to treatment.

In addition, its structure allows the set-up of ad hoc studies at different follow-up points in order to answer future research questions, such as genetic determinants of cirrhosis, HCV treatment response and tolerance to new antiretroviral treatments.

The schedule of follow-up visits in the cohort is based on recommended clinical practices [20], which facilitates the adherence of physicians and participants to the project and improves the management of patients.

One of the strengths of the cohort is also data collection, which is standardized, and includes all relevant data related to HIV and HCV infections to date. Frozen blood samples are regularly collected and centralized for planned and future studies (at enrolment, 92% of our patients had frozen blood samples). Lastly, data are systematically monitored and centralized in a single database.

The ANRS CO 13 HEPAVIH cohort however does have certain weaknesses, including the limited geographical distribution of participating centres, the nonparticipation of HIV-HCV patients from community-based clinics or primary care centers (although an uncommon feature of HIV-HCV care in France) and the fact that it is a prevalent cohort with a potential for selection of individuals with more severe disease and then at high risk of developing the outcomes of interest shortly after being recruited.

Patients lost to follow-up are of major concern in prospective cohorts. This might bias findings if attrition is linked to disease severity and/or death. To address this possible source of bias, the participating clinics together with the coordination centre have been providing sustained efforts to actively look for the patients who have not been seen for more than two years (10% of the overall cohort for the time being) and to encourage them to attend at least one more a follow-up visit. Finally, an information campaign has been performed, targeting patients and health care providers to disseminate the first study results and explain once again the importance of this study component.

## Conclusions

Prospectively designed large scale observational cohorts, representative of specific populations such as HIV-HCV co-infected patients, will be critical to address future clinical and public health questions related to chronic diseases of infectious origin. Nation-wide cohorts such as the French ANRS CO 13 HEPAVIH cohort may contribute to set the base for further broader collaborations to explore rare outcomes and long term prognosis.

## Competing interests

The authors declare that they have no competing interests.

## Authors' contributions

Conception and design: DS, FD, BS, JI, PS, LS, YB

Provision of study material or patients: MM, MAV, IPM, DN, PhB, ER, KB, PM, KL, AG, FR, ABS, CLC, DV, CG, CD

Collection and/or assembly of data : LM, SG, JD

Data analyses and interpretation : EP, FBS

Manuscript writing: MAL, PC, MW

All authors read and approved the final manuscript.

## Pre-publication history

The pre-publication history for this paper can be accessed here:

http://www.biomedcentral.com/1471-2334/10/303/prepub
